# Supplementary material for: Mummified precocial bird wings in mid-Cretaceous Burmese amber
Source: Nat Commun. 2016 Jun 28;7:12089. doi: 10.1038/ncomms12089 (PMC4931330; doi:10.1038/ncomms12089)
Supplement: Supplementary Information — Supplementary Figures 1-15, Supplementary Table 1, Supplementary Discussion and Supplementary References [file ncomms12089-s1.pdf]

Supplementary Figures

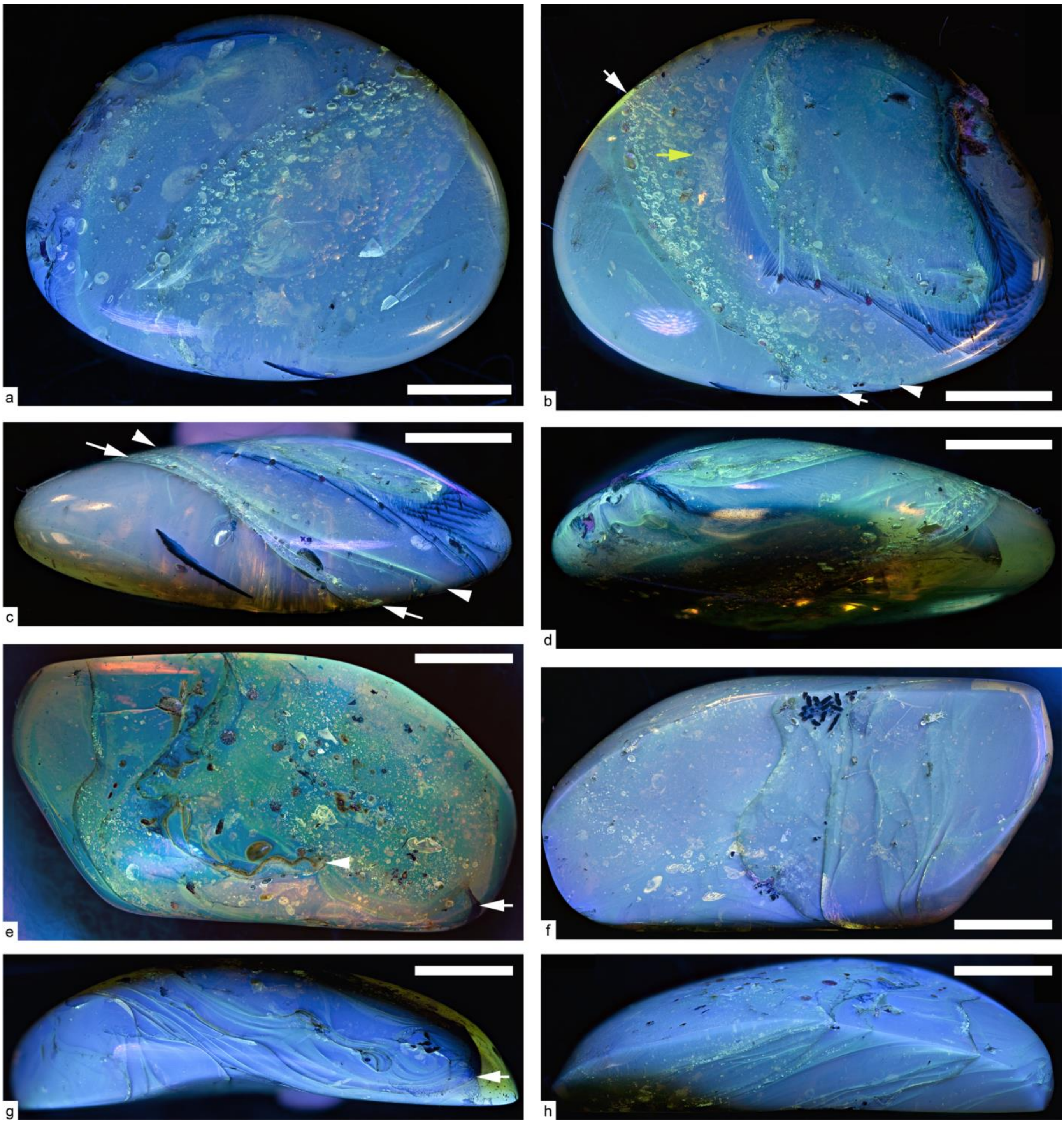

Supplementary Figure 1. UV photomicrographs of DIP-V-15100 (a–d) and DIP-V-15101 (e–h). a. Ventral view of wing, comparable to Fig. 2j, Supplementary Fig. 2d. b. Dorsal view of wing, comparable to Figs. 2a, g, h, Supplementary Fig. 2a. White arrows mark a major drying line, arrowheads mark a minor drying line, and yellow arrow indicates claw mark within drying line. c. Posterior view of wing, comparable to Figs. 2b, c. Arrows mark major drying line, arrowheads mark minor drying line. d. Anterior view of wing, comparable to Fig. 2i. e. Ventral view of wing prior to trimming amber piece, generally comparable to Fig. 3f, Supplementary Fig. 4. Apex of wing breaches amber surface in lower right corner of image (arrow), and skin flap breaches amber surface near middle of specimen. (Arrowhead marks downy anterior corner of skin flap, used as a reference point in Supplementary Fig. 4). f. Dorsal view of wing prior to trimming amber piece, generally comparable to Fig. 3a, Supplementary Fig. 3a. Black cylindrical objects near middle of upper edge of specimen are frass pellets exposed at the amber surface. g. View toward anterior margin of wing, with exposed primary remiges and the minor flow lines that they create in surrounding amber indicated by arrow. Exposure corresponds to lower margin in e, and region of feather exposure is similar to Fig. 3g. h. View toward posterior margin of wing, corresponding to upper margin in e. Scale bars 5 mm in a–d; 1 cm in e–h.

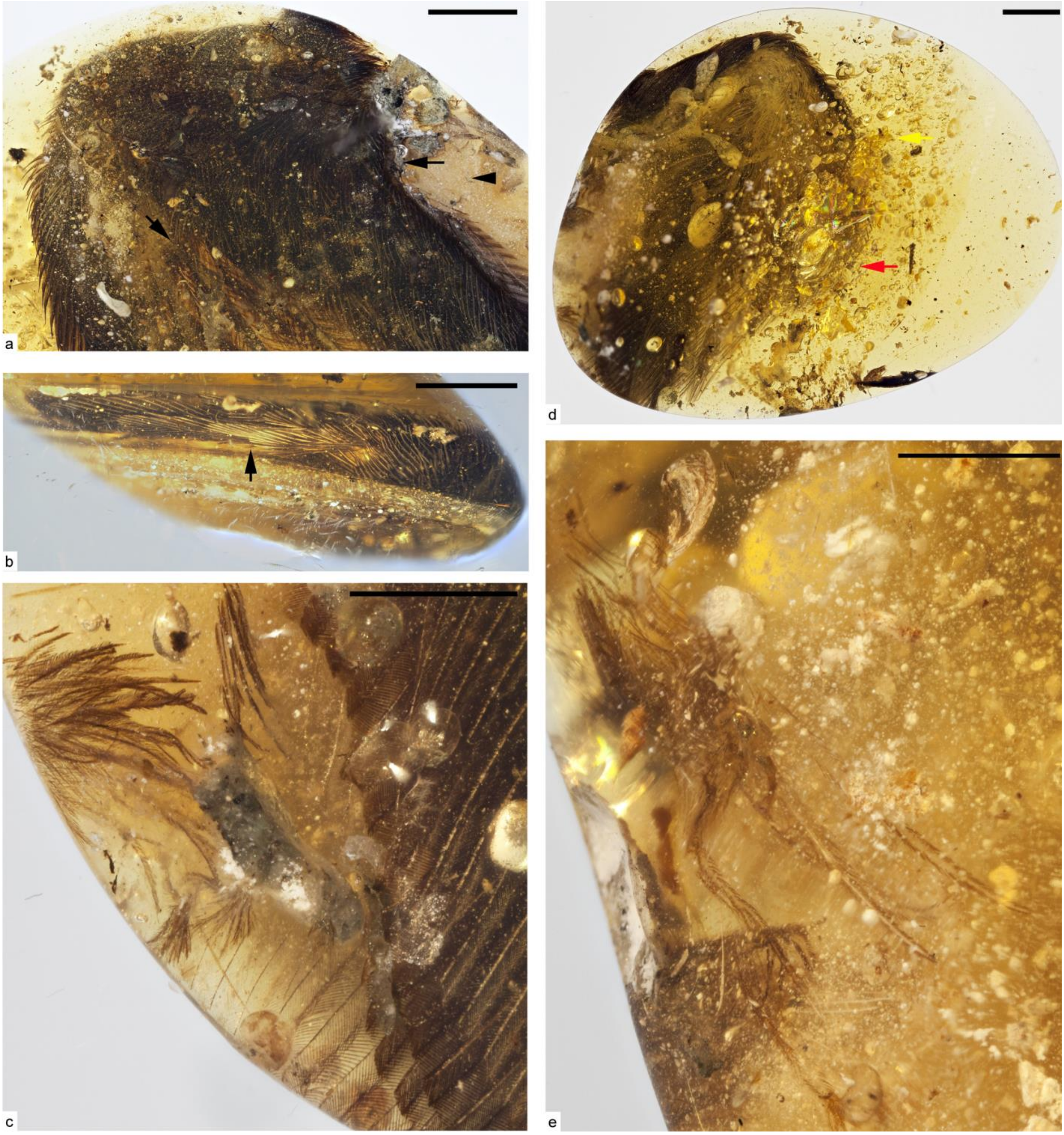

Supplementary Figure 2. Additional photomicrographs of DIP-V-15100. a, Overview of anterior margin of wing, with poorly concealed bases of primary remiges (inclined arrow), and prevalence of milky amber adjacent to region where radius and ulna are truncated (arrowhead), as opposed to the mottled grey clay that was sampled for SEM analysis (horizontal arrow). b. Anterior/apical view of wing, highlighting patch of pale feathers at base of alula (arrow), and position of wing between flow lines in amber. c. Ventral view of wing, near base (lower left margin of d), showing remiges truncated at amber surface, as well as a tufts of free-floating contour feathers that may stem from either the wing or body regions. d. Ventral overview of wing, highlighting decay products in amber, as well as the position of visible claws (red arrow), or struggle marks in amber flow lines (yellow arrow), shown in greater detail in Fig. 2k. e. Isolated contour feathers with variation in pigmentation, possessing pale rami and basal barbule sections, as opposed to the monotone contours in c. Scale bars 2.5 mm in a, b, d; 1 mm in c, e.

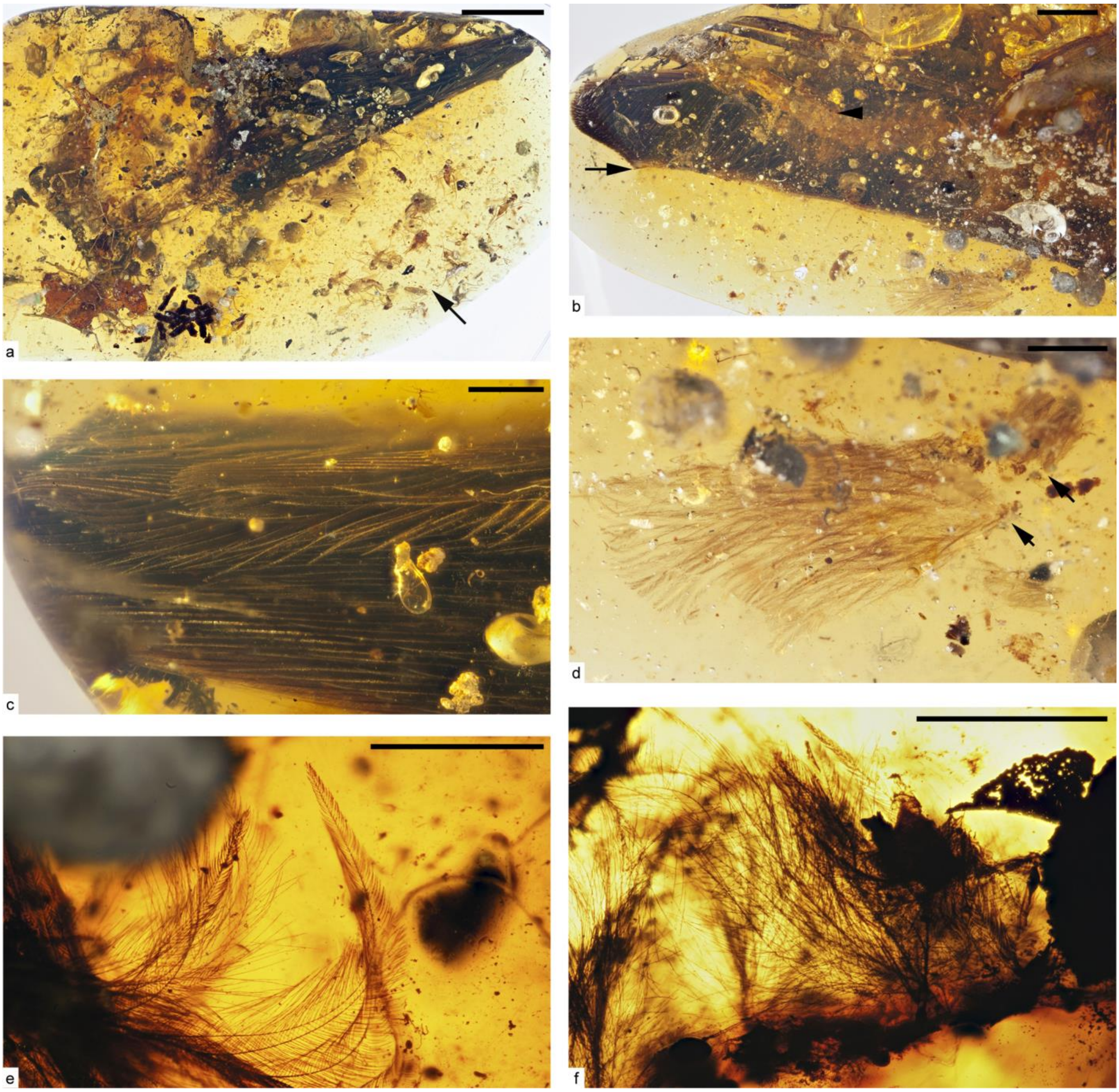

Supplementary Figure 3. Additional photomicrographs of DIP-V-15101. a. Overview of dorsal surface of wing, prior to trimming to remove swarm of dipterans (arrow) that impeded views. b. Overview of ventral surface of wing, showing curled posterior margin of remiges (arrow) due to strong interactions with resin flows, as well as veil of milky amber that obscures ventral view of manus (arrowhead). c. Apical view of flight feather mass, displaying range of pigmentation within each feather, and paler central portions of each barb. d. Clusters of pale contour feathers that may retain original insertions arrangement because of skin fragment preservation. Positioned posterior to wing margin in b. e. View of pale contour feathers differentiated into plumulaceous bases (background) and pennaceous apices with higher pigment densities (foreground) near skin flap (compound microscopy, t.l.). f. Definitive down inserting into partially carbonized section of skin flap examined in Supplementary Fig. 4 (compound microscopy, t.l.). Scale bars 2.5 mm in a, b; 1 mm in c–f.

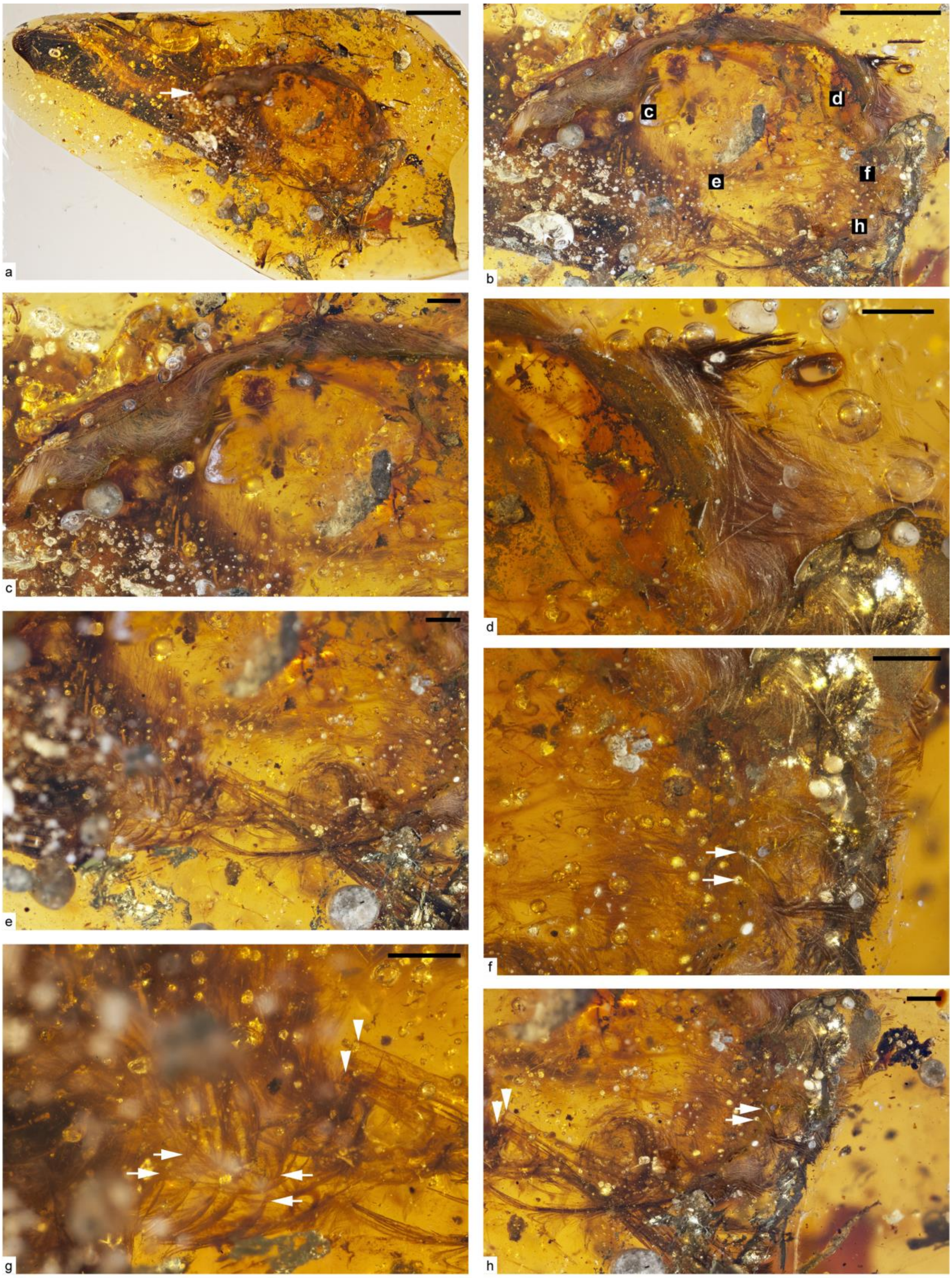

Supplementary Figure 4. Details of skin flap preserved in DIP-V-15101. a. Overview of ventral surface of wing, with proximal end of skin flap positioned near arrow. b. Reference diagram for subsequent images of skin flap (letters correspond to figure components below, and are positioned within concave surface created by skin flap). c. Proximal extent of skin flap, with mat of white or extremely pale downy feathers compressed by interaction with a drying line toward top of figure. d. Mottled contour feathers that range from nearly white (right side of image) to dark brown in colour (left side of image). Skin is either partially carbonized in this position, or mineral growth has occurred along the same plane as original skin tissue (lower left corner of image). e. Tangled mass of contour feathers, down, and isolated flight feathers (detailed in g). f. Bases of contour feathers preserved *in situ* within skin sheet (arrows indicate two examples of a widespread pattern), demonstrating a range of plumage colours from dark brown pennaceous barbules interspersed with white plumulaceous barbules (right side of image) to pale plumulaceous or reduced pennaceous barbules (left side of image). g. Details of contour feathers showing original arrangement into feather follicle rows (arrows), alongside the bases of isolated flight feathers (arrowheads). h. Position of isolated flight feathers (arrowheads) with respect to skin sheet with in situ feathers (arrows). Scale bars 2.5 mm in a, b; 1 mm in c–h.

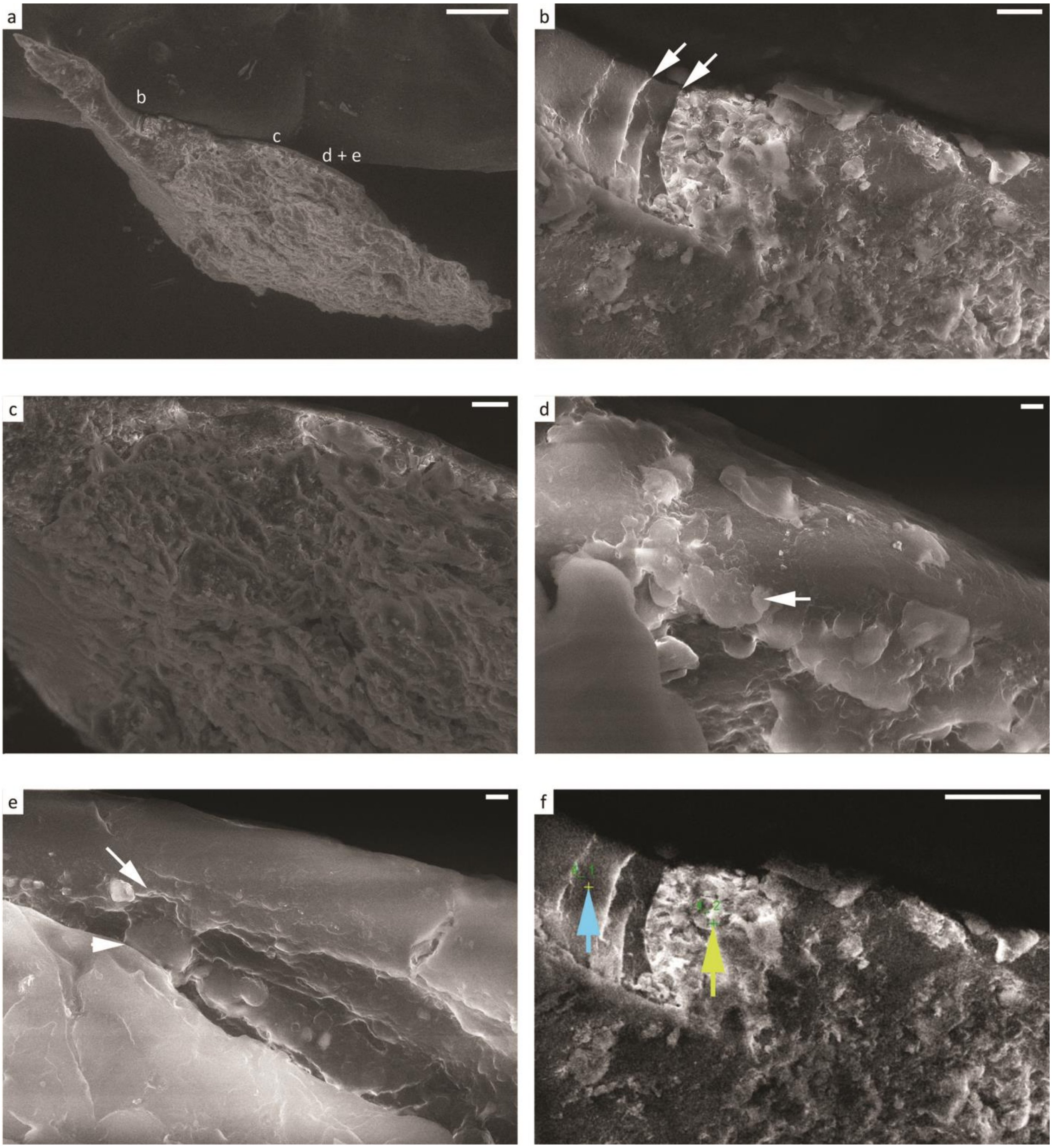

Supplementary Figure 5. SEM images of flake of surficial amber from DIP-V-15100. a. Overview with letters indicating positions of detailed images. Upper surface of specimen consists of thin amber rind, with narrow and recessive layer of carbonized material below amber, and then large area occupied by clay minerals. b. Detail of feather rachis or ramus breaching surface of flake adjacent to amber (arrows mark edges of cylindrical carbon film). c. Overview of platy clay minerals that have filled void between feather layer and radius and ulna. d. Detail of partial pyrite framboids (arrow) along lower margin of amber rind. e. Detail of recessive area between amber rind and clay infill, where feather layer is visible in hand specimen (arrows mark edges of recess). f. Detail of area sampled for EDS analysis with regions of interest marked by arrows and corresponding to Supplementary Fig. 6. Blue arrow marks region of interest within carbon film of rachis or ramus. Green arrow marks region of interest within clay infill of pith cavity. Scale bars 10 µm in a; 20 µm in b, f; 1 µm in c–e.

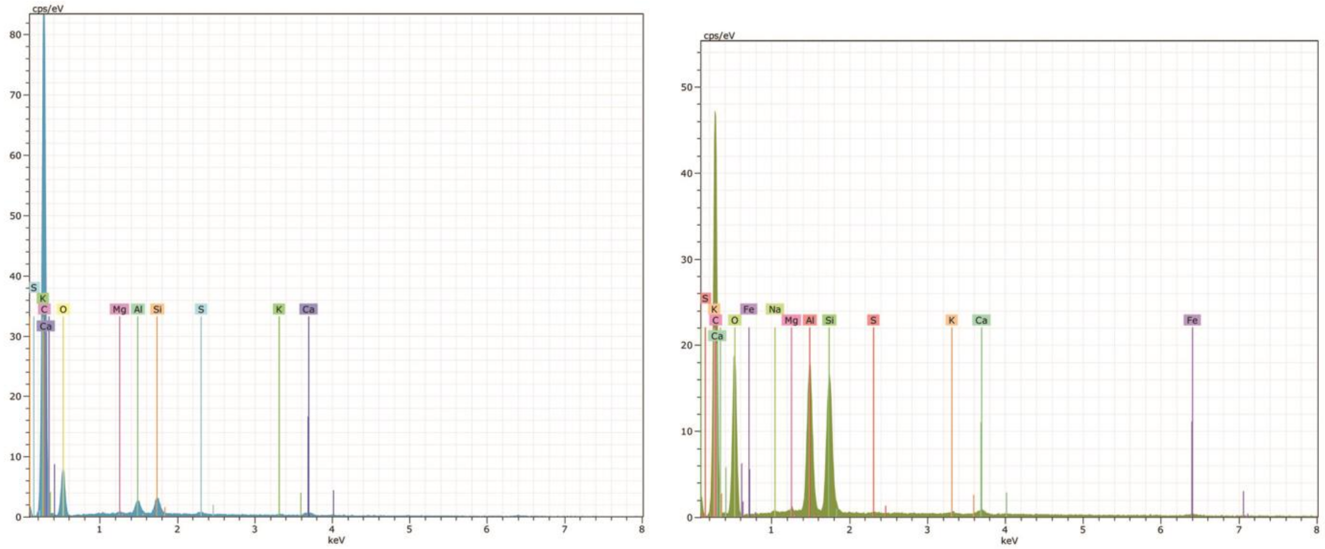

El	AN	Series	unn. C [wt.%]	norm. C [wt.%]	Atom. C [at.%]	Error (1 Sigma) [wt.%]
C	6	K-series	76.61	76.61	81.92	8.70
O	8	K-series	21.50	21.50	17.26	3.06
Si	14	K-series	0.69	0.69	0.32	0.06
Al	13	K-series	0.64	0.64	0.31	0.06
Ca	20	K-series	0.31	0.31	0.10	0.04
S	16	K-series	0.12	0.12	0.05	0.03
K	19	K-series	0.07	0.07	0.02	0.03
Mg	12	K-series	0.05	0.05	0.03	0.03
		Total:	100.00	100.00	100.00	

a

El	AN	Series	unn. C [wt.%]	norm. C [wt.%]	Atom. C [at.%]	Error (1 Sigma) [wt.%]
C	6	K-series	57.12	57.12	66.24	6.76
O	8	K-series	33.33	33.33	29.01	4.24
Al	13	K-series	4.74	4.74	2.45	0.26
Si	14	K-series	4.06	4.06	2.01	0.20
Ca	20	K-series	0.25	0.25	0.09	0.04
Fe	26	K-series	0.21	0.21	0.05	0.04
Mg	12	K-series	0.10	0.10	0.06	0.03
Na	11	K-series	0.07	0.07	0.04	0.03
K	19	K-series	0.07	0.07	0.03	0.03
S	16	K-series	0.05	0.05	0.02	0.03
		Total:	100.00	100.00	100.00	

b

Supplementary Figure 6. EDS results from DIP-V-15100 SEM observation, examining regions of interest specified in Supplementary Fig. 5. a. EDS peaks and semi-quantitative elemental analysis for ROI within carbon film of rachis or ramus. b. EDS peaks and semi-quantitative elemental analysis for ROI within clay infill of rachis or ramus.

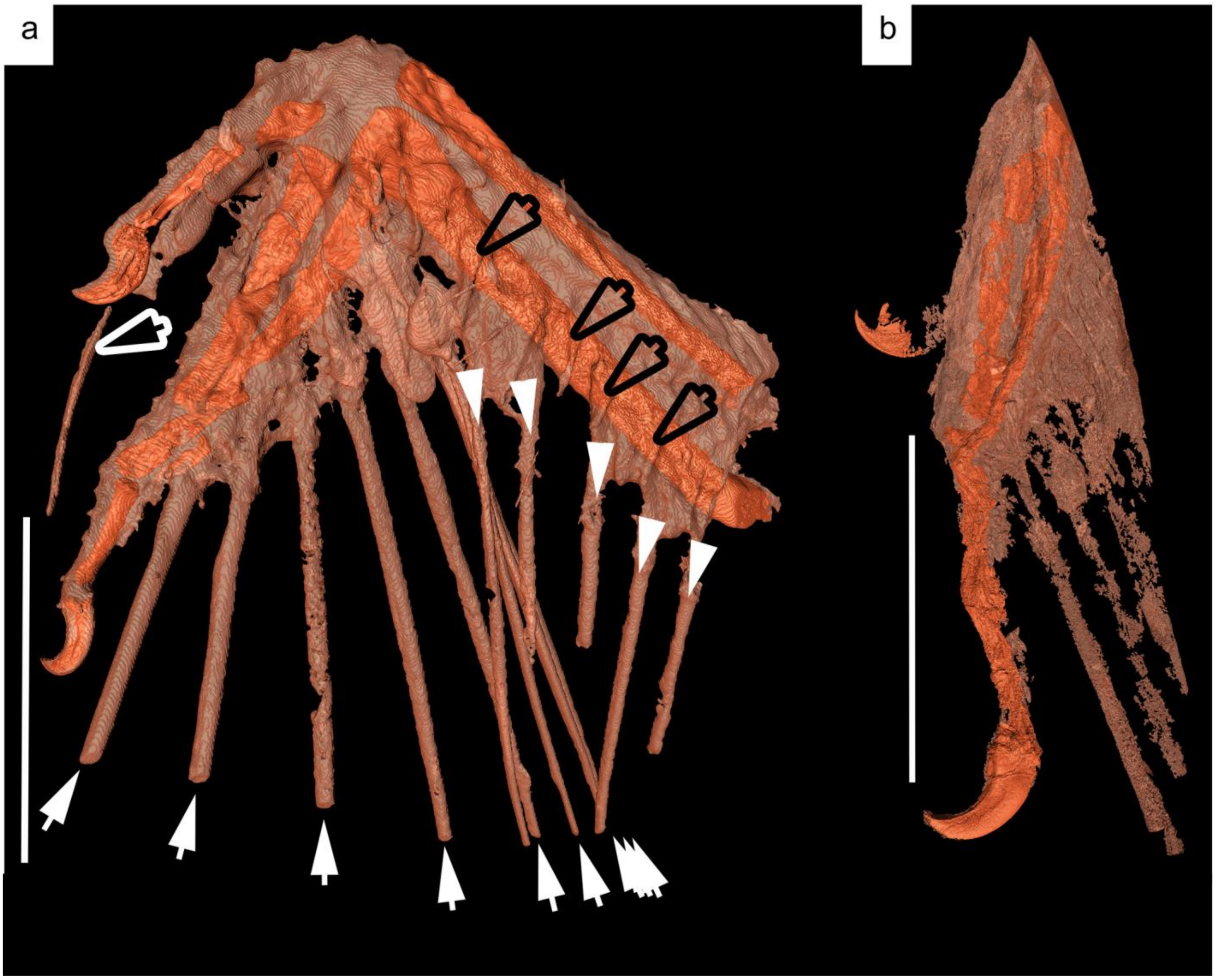

Supplementary Figure 7. SR x-ray µCT reconstructions of DIP-V-15100 and DIP-V-15101, with bone outlines overlaying soft tissue and feather attachments depicted in Fig. 1. a. DIP-V-15100 with solid white arrows indicating primary feathers (attached to manus), solid white arrowheads indicating secondary feathers (attached to ulna), empty black arrows indicating bases of coverts, and empty white arrow indicating base of alular feather. b. DIP-V-15101 showing attachments of primary feathers to manus. Membrane anterior to radius is prepatagium, surrounding alula is alular patagium, membrane posterior to ulna is postpatagium, and swellings within postpatagium correspond to calami in feathers. Scale bars 5 mm.

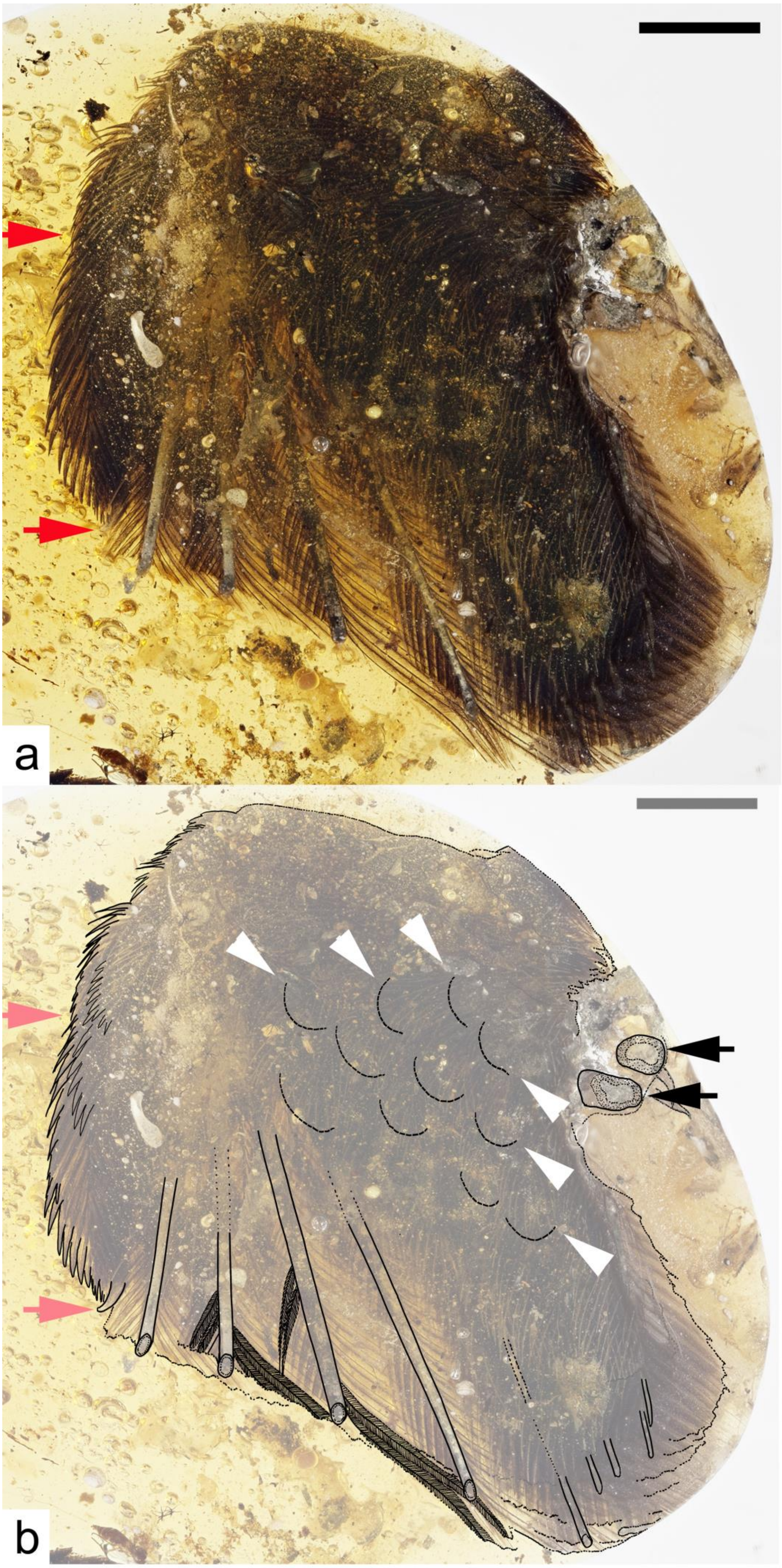

Supplementary Figure 8. Expanded version of Fig. 2a, with interpretive diagram of plumage in DIP-V-15100. a. Expanded version of Fig. 2a. Red arrows correspond to positions of claws. b. Interpretive diagram highlighting position of covert feather rows (hatched lines indicate approximate positions of feather apices, in rows between white arrowheads); with position of exposed radius and ulna indicated with stippled section outlines on right side of figure, as well as translucent margin of enveloping soft tissue; margins of alular feathers marked with detailed outlines of barbs on left side of figure; positions of most visible primary feathers outlined, and barb asymmetry indicated at lower margin of wing; details of primary feather structure examined in Supplementary Fig. 9. Scale bars 2.5 mm.

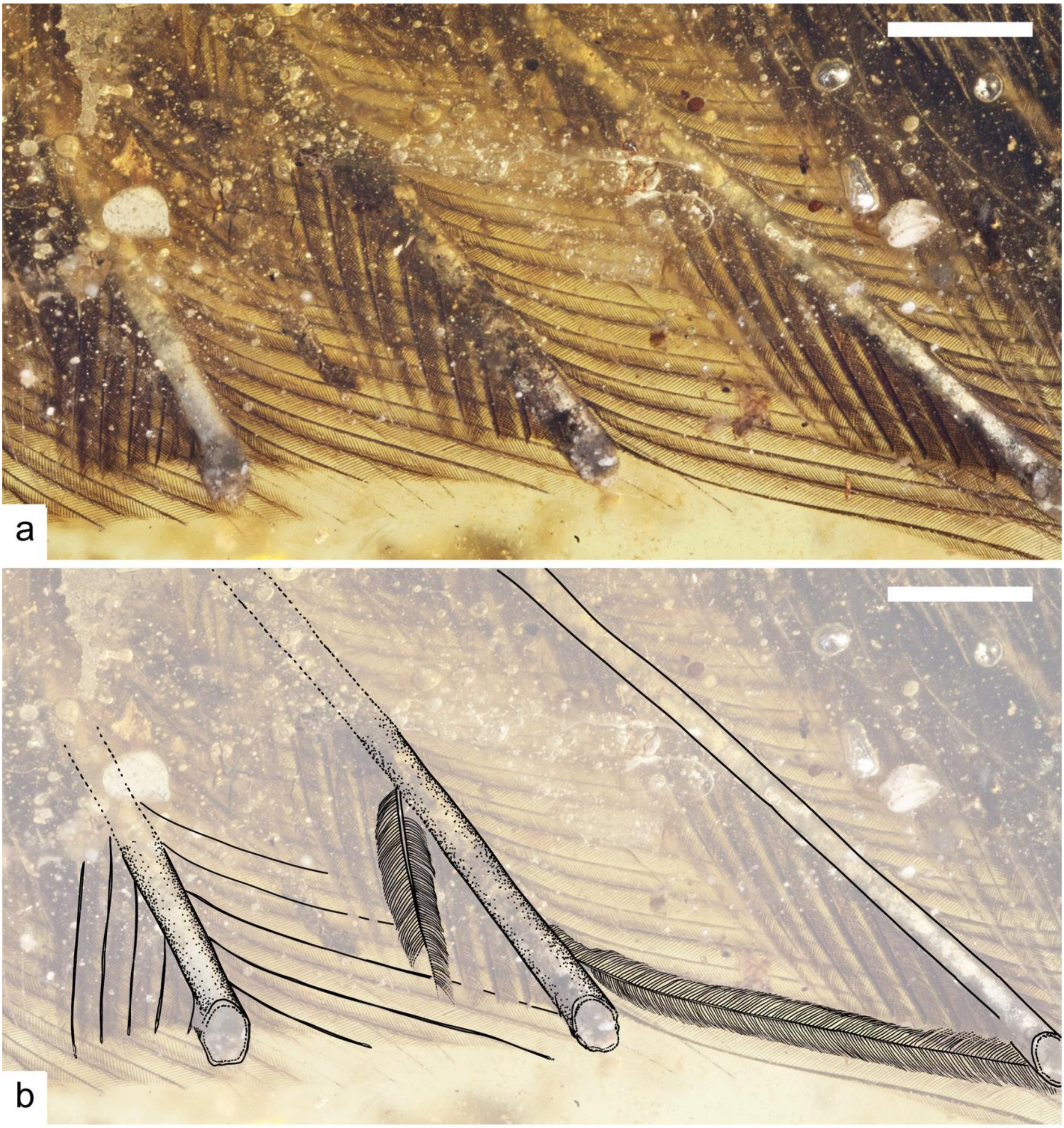

Supplementary Figure 9. Expanded version of Fig. 2b, with interpretive diagram for primaries in DIP-V-15100. a. Expanded version of Fig. 2b. b. Interpretive diagram highlighting rachis outlines, where they are cut at various angles by the polished amber surface, as well as some examples of barb rami positions on rachises, and barbule positions on rami. Scale bars 1mm.

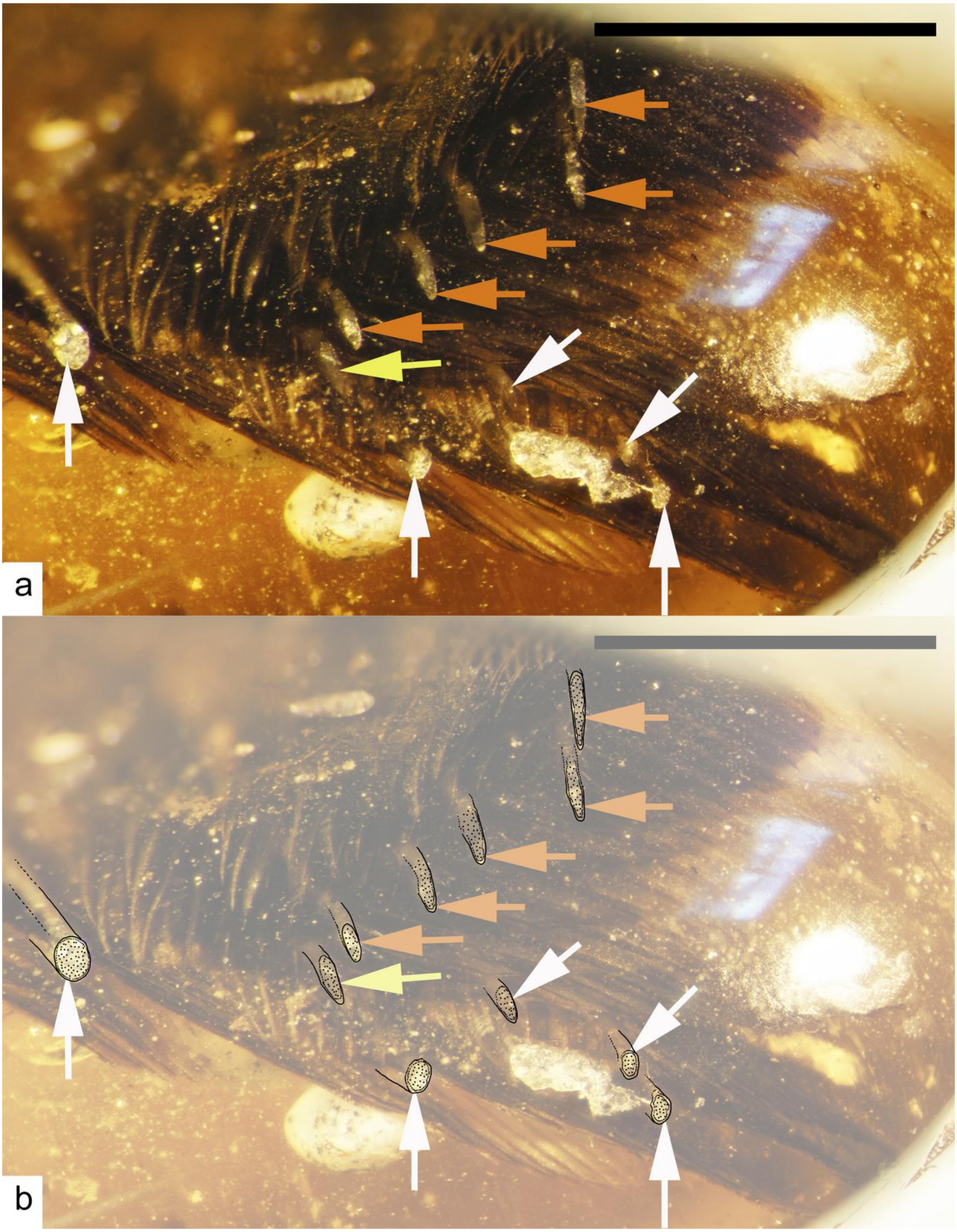

Supplementary Figure 10. Expanded version of Fig. 2c, with interpretive diagram for region of primary and secondary feather overlap in DIP-V-15100. a. Expanded version of Fig. 2c (white arrows point to primaries, orange arrows point to secondaries, yellow arrow points to indeterminate feather). b. Interpretive diagram highlighting points where each rachis breaches amber surface, with pith cavities in each rachis stippled to indicate angle of section created by polished surface of amber. Secondary feathers near upper right of image sectioned obliquely. Scale bars 2.5 mm.

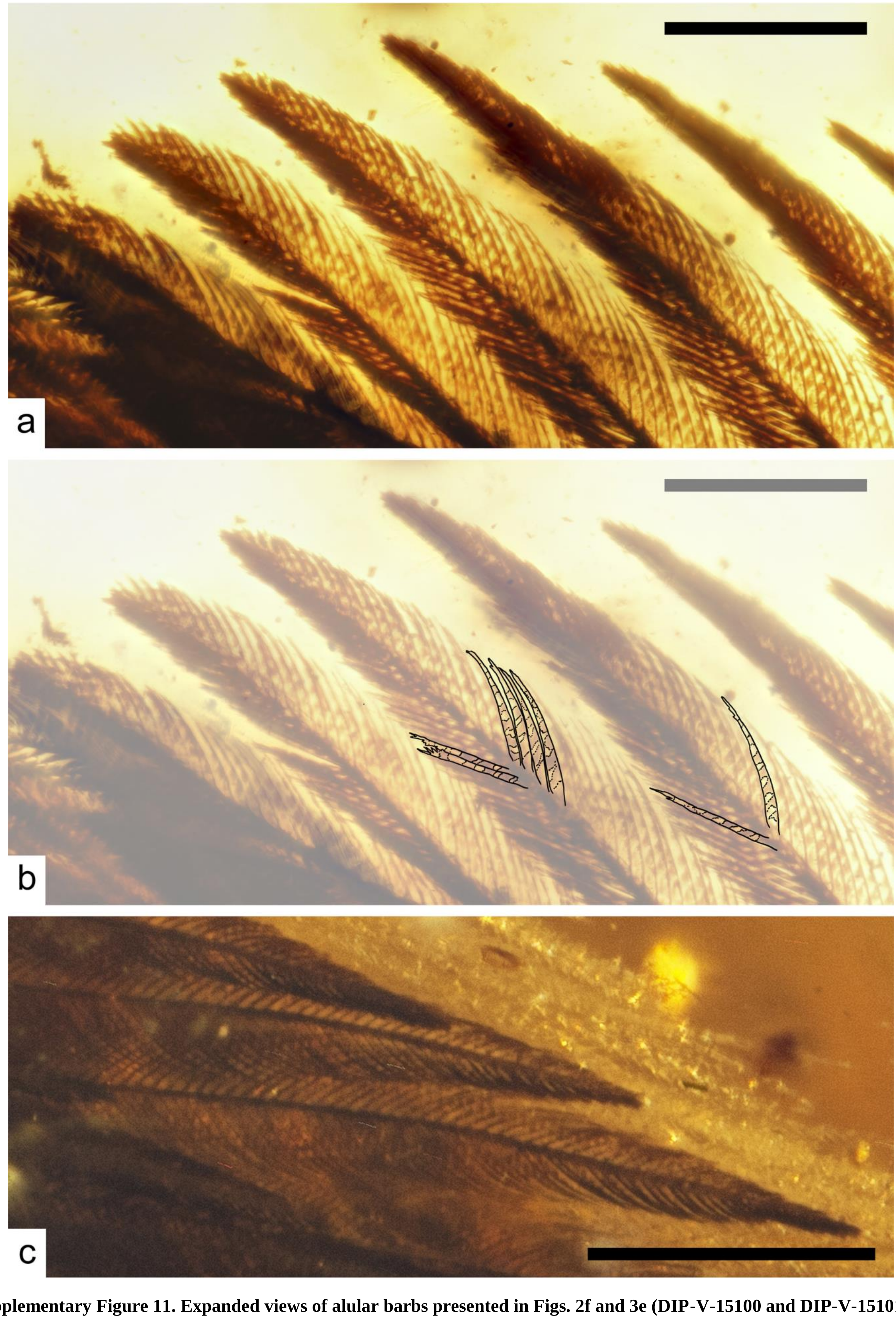

Supplementary Figure 11. Expanded views of alular barbs presented in Figs. 2f and 3e (DIP-V-15100 and DIP-V-15101, respectively). a. Expanded version of Fig. 2f, alular barbs in DIP-V-15100. b. Interpretive diagram highlighting barbule structure and pigmentation banding within basal cells of blade-like barbules. Proximal barbules are on right side of each barb, and distal barbules are on left side of each barb in this view. c. Expanded view of Fig. 3e, heavily pigmented alular barbs in DIP-V-15101 in foreground, with translucent barbules from ventral plumage in background (upper right). Scale bars 0.25 mm in a, b; 0.5 mm in c.

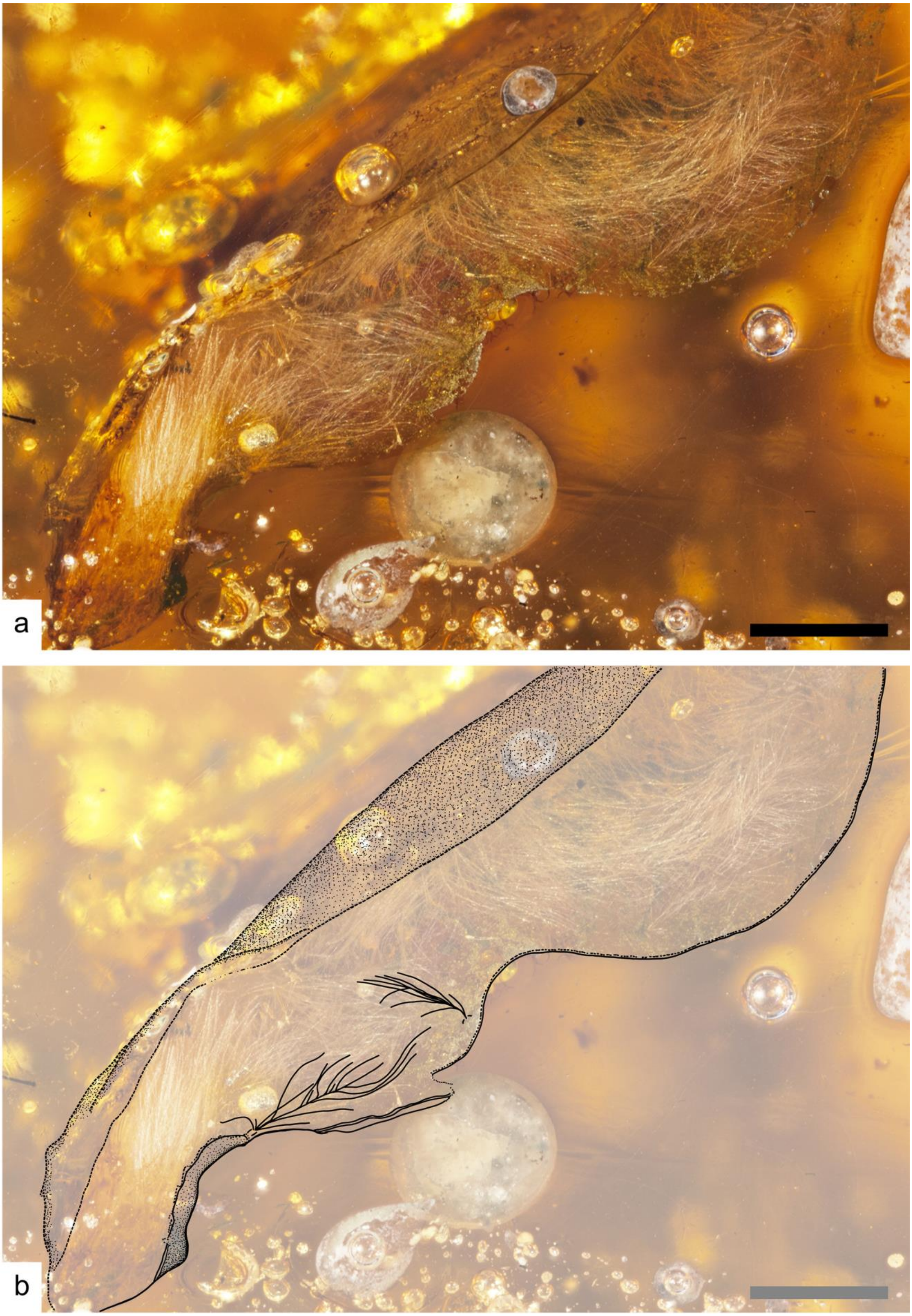

Supplementary Figure 12. Expanded views and interpretive diagram of skin flap and plumage presented in Fig. 3j, and detailed in Supplementary Fig. 4, specimen DIP-V-15101. a. Expanded version of Fig. 3j, skin flap with dense mat of pale, downy feathers. b. Interpretive diagram, showing flow line within amber that has compacted the plumage (stippled upper surface), general outlines of two downy feathers that are visible among the dense plumage, and the translucent sheet of preserved skin (lower surface in diagram, with stippling toward lower left indicating region of partial carbonization or thicker tissue). Scale bars 1 mm.

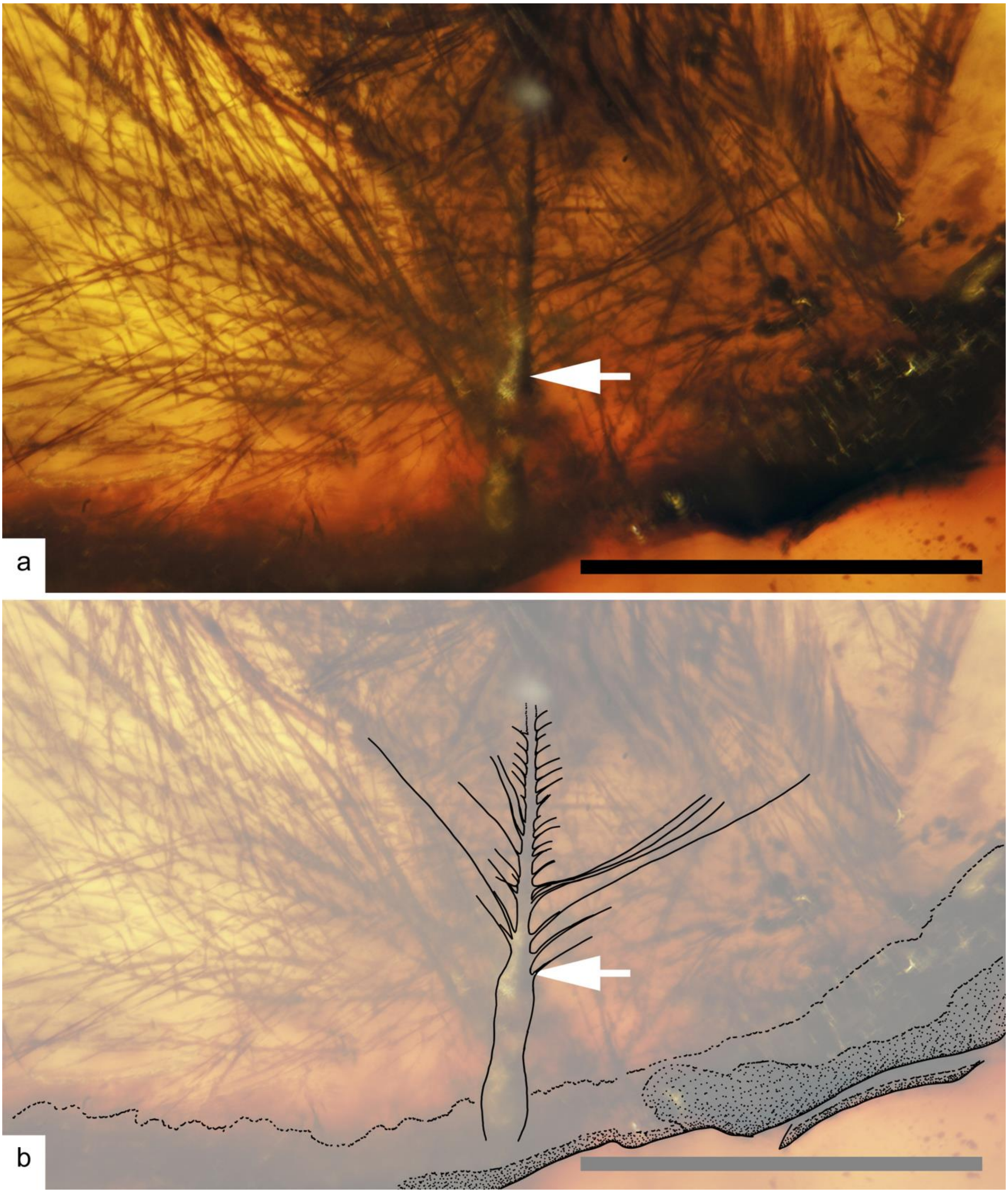

Supplementary Figure 13. Expanded view and interpretive diagram of down feather base and insertion into skin, specimen DIP-V-15101. a. Expanded version of Fig. 3k, with arrow indicating calamus, small basal sheath, and flap of preserved skin. b. Interpretive diagram highlighting barbule bases on rachis, and outline of translucent skin sheet. Stippled areas indicate partial carbonization or thicker regions within tissue, while upper surface of skin (toward top of figure) remains difficult to discern due to translucency. Scale bars 0.5 mm.

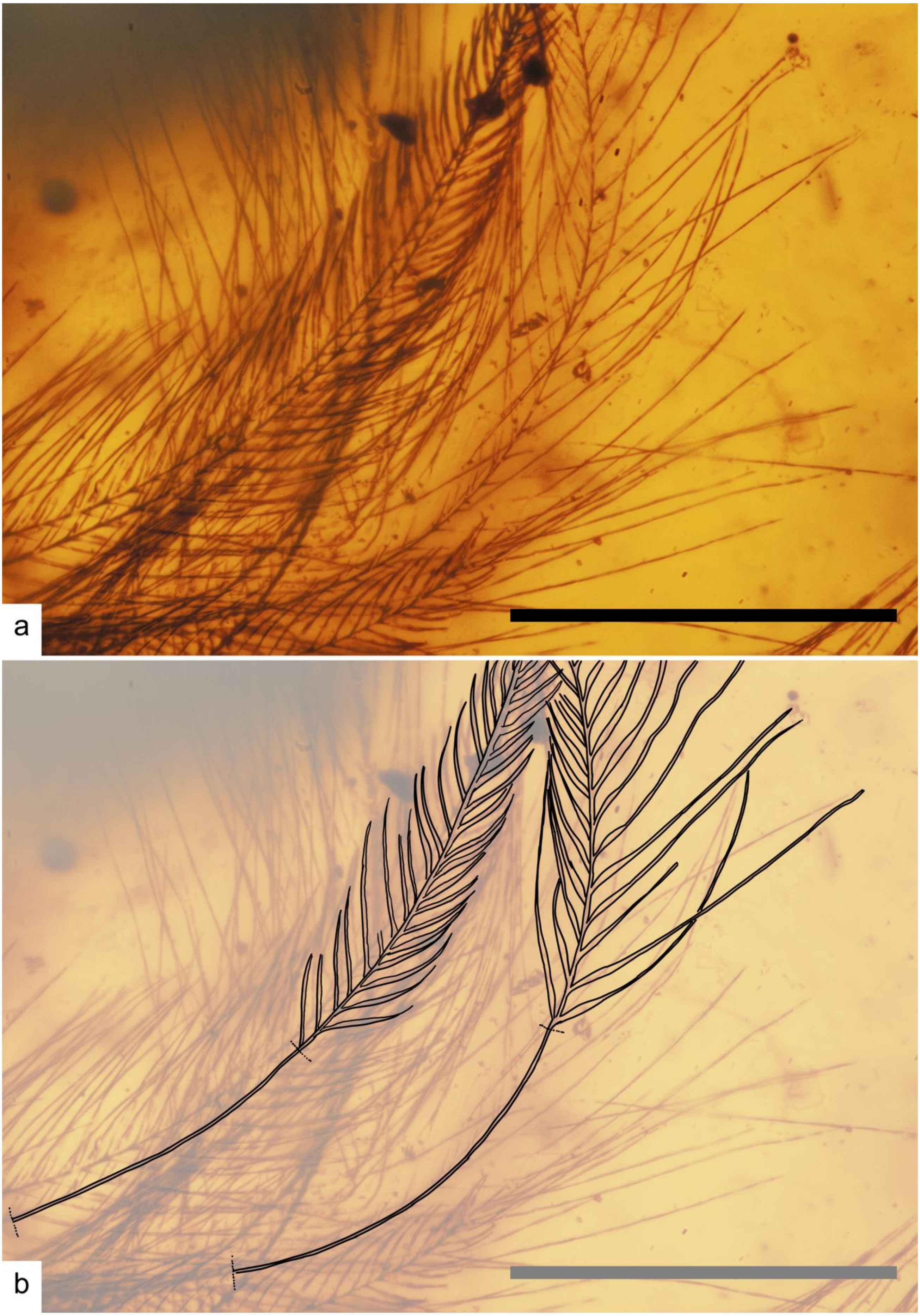

Supplementary Figure 14. Expanded view and interpretive diagram of contour feather barbs, from feathers preserved on skin flap of specimen DIP-V-15101. a. Expanded version of Fig. 3l, region where plumulaceous barbules from base of contour feather overlap with pennaceous barbules from apical part of contour feather. b. Interpretive diagram highlighting barbule outlines for one pennaceous and one plumulaceous barb from the same feather. Open-ended barbules in illustration had unclear apical portions, and region between dashed lines for each barb shows simplified trace of barb ramus outline. Scale bars 0.5 mm.

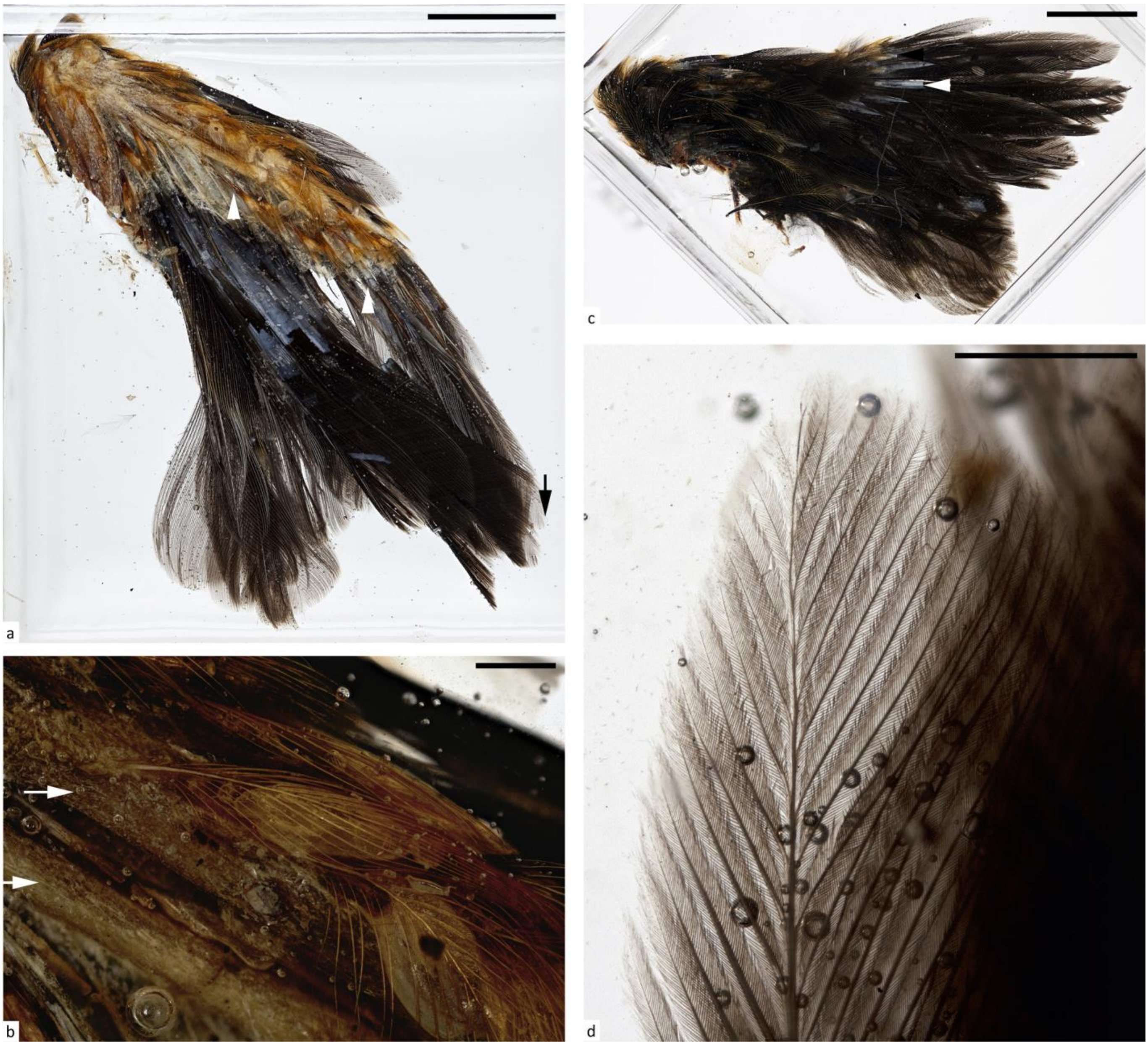

Supplementary Figure 15. Photomicrographs of modern robin (*Turdus migratorius*) wings from partially decayed juvenile specimen. a. Ventral view of left wing, cut through radius and ulna, with partially sheathed contour and flight feathers (arrowheads). b. Detail of apterium in a, with contour feather insertion and translucent skin covering bones of metacarpus (arrows). c. Dorsal view or right wing, cut through radius and ulna, with partially sheathed primary flight feathers (arrowheads). d. Detail of barbule structure and pigmentation in primary flight feather (positioned near black arrow in a). Scale bars 10 mm in a, c; 1 mm in b, d.

Supplementary Tables

Supplementary Table 1 | Measurements of DIP-V-15100 and DIP-V-15101 (in mm).

**Elements**	**Abb.**	**DIP-V-15100**		**DIP-V-15101**	
		Length	Width	Length	Width
Ulna	ul	7.94	0.67	—	—
Radius	ra	6.50	0.33	—	—
Alular metacarpal	am	1.00	0.50	—	—
Major metacarpal	mam	4.17	0.50	4.95	0.33
Minor metacarpal	mim	4.72	0.56	6.24	0.43
Alular phalanx-1	al-1	1.83	0.28	2.95*	0.21
Alular phalanx-2	al-2	1.28	0.47	1.81	0.71
Major phalanx-1	ma-1	2.22	0.33	3.10	0.19
Major phalanx-2	ma-2	1.78	0.25	2.57	0.24
Major phalanx-3	ma-2	1.28	0.47	2.14	0.90
Minor phalanx-1	mi-1	1.00	0.22	1.38	0.24

*probably including part of alular metacarpal.

Supplementary Discussion

Commentary on feather types not represented in the amber samples. The two wing samples each display a suite of feathers that are very similar to those of modern birds, conforming to the evolutionary-developmental Stage V of Prum[1]. Down, contour, and asymmetrical vaned flight feathers of modern aspect were all clearly represented within each sample. We were not able to observe an afterfeather among any of the wing feathers. This may be due to the extent of basal overlap within the plumage, pale colouration of afterfeathers in the surrounding amber, or their true absence. Where cross sections were formed through the rachises of larger flight feathers in DIP-V-15100 some specimens appear to have a shallow ventral groove (Fig. 2d): this may hint at the presence of an afterfeather, but it is not conclusive. We are reluctant to assign significance to this perceived absence until a wider range of samples show a comparable pattern.

Some of the particulate matter trapped within the amber may represent powders produced by feathers such as powder downs, but it is not possible to examine structures that are only one micron in diameter within the current sample set (which is the scale of down or feather powders[2]). Similarly, some of the darkly stained surfaces present within each amber piece may represent oils from the plumage, but it is exceedingly difficult to differentiate between oils, decay products, and oxidized amber flow lines without destructive chemical analyses.

Comparison to modern *Turdus migratorius* (juvenile robin) embedded wings. The small size of the wing section preserved in each amber specimen, coupled with their immature osteological features suggested that they may be from juveniles. In order to compare the amber samples to relevant modern material, we epoxy embedded sections taken from modern robin wings. These specimens were from the partially decayed remains of an immature robin. Unlike the amber samples, plumage in the modern material clearly displayed sheathed feathers (Supplementary Fig. 15). Even though the skin in the modern samples had partially dried, it too became translucent when embedded in a synthetic resin. Feather oils coloured some of the bubbles within the epoxy blocks, but not to the same extent as the decay-related bubbles inferred in DIP-V-15100. The robin did not undergo significant anaerobic decay while embedded in the synthetic resin, and consequently did not have a chance to develop the waxy adipocere preservation that is inferred for the apterium of DIP-V-15100. Embedding imparted a slightly darker apparent overall plumage colour to the robin wing, but photomicrographs of the individual barbs within the primary flight feathers (Supplementary Fig. 15d) still provide a clear indication of original colour and pigmentation intensity. The latter observation supports inferences regarding the original colours of the amber-embedded wings.

Supplementary References

1. Prum, R.O. Development and evolutionary origin of feathers. *J. Exp. Zool.* **285,** 291–306 (1999).

2. Lucas, A.M. & Stettenheim, P.R. *Avian Anatomy: Integument* (US Government Printing Office, Washington, 1979).
